# UbC-*StarTrack,* a clonal method to target the entire progeny of individual progenitors

**DOI:** 10.1038/srep33896

**Published:** 2016-09-22

**Authors:** María Figueres-Oñate, Jorge García-Marqués, Laura López-Mascaraque

**Affiliations:** 1 Instituto Cajal, CSIC, Madrid, Spain

## Abstract

Clonal cell analysis defines the potential of single cells and the diversity they can produce. To achieve this, we have developed a novel adaptation of the genetic tracing strategy, UbC-*StarTrack*, which attributes a specific and unique color-code to single neural precursors, allowing all their progeny to be tracked. We used integrable fluorescent reporters driven by a ubiquitous promoter in PiggyBac-based vectors to achieve inheritable and stable clonal cell labeling. In addition, coupling this to an inducible Cre-LoxP system avoids the expression of non-integrated reporters. To assess the utility of this system, we first analyzed images of combinatorial expression of fluorescent reporters in transfected cells and their progeny. We also validated the efficiency of the UbC-*StarTrack* to trace cell lineages through *in vivo*, *in vitro* and *ex vivo* strategies. Finally, progenitors located in the lateral ventricles were targeted at embryonic or postnatal stages to determine the diversity of neurons and glia they produce, and their clonal relationships. In this way we demonstrate that *UbC*-*StarTrack* can be used to identify all the progeny of a single cell and that it can be employed in a wide range of contexts.

In the brain, groups of clonally related cells are responsible for forming all the adult neural circuits. Thus, clonal analys1is of single cells is a powerful means to understand how neural cells acquire their identity and functional differences. There is currently much controversy regarding the commitment and heterogeneity of neural progenitors. Classical theory considered the radial glia as the main neural stem cell capable to generating all neural cell types^1^. More recently, other neural progenitors are thought to be committed to certain cell lineages and to generate distinct neural progeny at specific developmental times^2^. Thus, it is crucial to understand the broad heterogeneity of progenitor pool and to be able to distinguish the progeny of an individual progenitor from the rest of the cells in the brain.

Retroviral vectors carrying a single reporter gene were able to show that radial glia cells are indeed a common progenitor for both glial and neuronal cells^3^. In addition, this tool has been also used to elucidate clonal relationships between neurons^4^. Other clonal methods employed isolated recombination in stochastic cells involving different transgenic lines, as mosaic analysis with double markers^5^,^6^. However, since these approaches label a small number of clonally related cells, they are not entirely appropriate to analyze inter/intra-clonal relationships. To perform larger and more reliable clonal analyses, libraries of tagged retroviruses have been designed^7^. Alternatively, other approaches to identify clonally-related cells rely on somatic mutations during the DNA replication, associated with cell division^8^, or on genetic multicolored cell labeling using either transgenic animals or infection with fluorescent lentivirus^9^,^10^,^11^. Recently, multicolor non-viral PiggyBac transposon mediated genomic integration has proved to be a very useful tool to define progeny at the single cell level^12^,^13^,^14^ Indeed, we previously designed the *StarTrack* strategy in order to study clonally related astroglial cells that are derived from single progenitors using the hGFAP promoter^15^,^16^. Here we present a novel adaptation of that system, UbC-*StarTrack*, to perform clonal analysis and tracing of neural cell types derived from single progenitors. Unlike other lineage approaches, our ubiquitous lineage-tracing method avoids the expression of non-integrated copies of electroporated plasmids, taking advantage of the Cre-lox strategy to achieve unequivocal labeling of the entire progeny of single progenitors. Indeed, the strategy behind this method represents an exciting tool for other areas in the field of biomedicine, avoiding the use of genetically engineered mouse models.

## Results

### UbC-*StarTrack* multicolor labeling for single-cell clonal tracking

**Figure 1:**
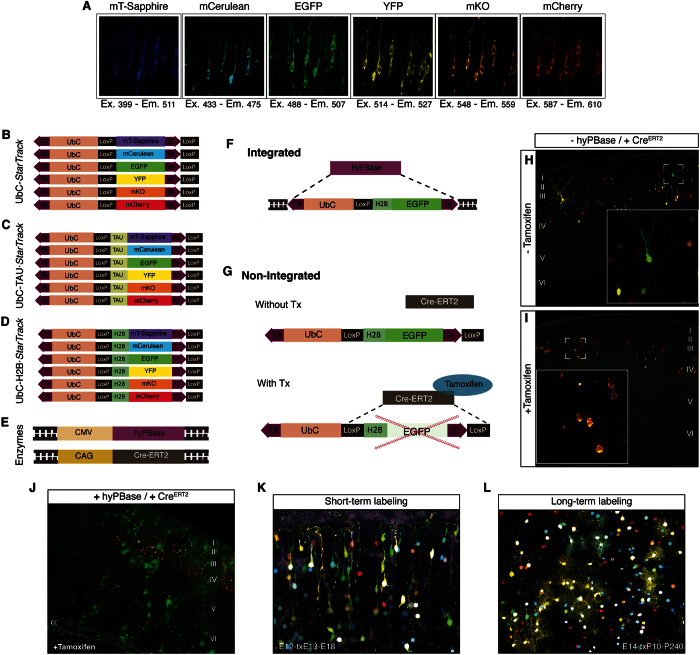
UbC-*StarTrack* design. (**A**) Fluorescent reporters: mT-Sapphire, mCerulean, Enhanced green fluorescent protein (EGFP), Yellow fluorescent protein (YFP), monomeric Kusabira Orange (mKO) and mCherry. Spectral profiles for the single fluorophores: Ex, Major excitation peak; Em, Major emission peak. (**B**) Scheme of the six ubiquitous constructs with the different fluorescent proteins expressed in the cytoplasm: UbC-*StarTrack*. (**C**) Scheme of the six constructs with the different TAU-fluorescent fusion proteins: UbC-TAU·StarTrack. (**D**) Scheme of the six ubiquitous constructs encoding the different H2B-fluorescent fusion proteins: UbC-H2B·StarTrack. (**E**) Hyperactive version of the PiggyBac transposase (hyPBase) and an inducible Cre recombinase (Cre-ER^T2^) were added to the UbC-*StarTrack* electroporation mixture. Ubiquitous promoters drove the expression of both constructs. (**F**) hyPBase introduces several copies of constructs into the cell genome after electroporation. (**G**) Cre recombinase activated by tamoxifen (Tx) deletes the episomal copies of vectors (not integrated by the transposase). (**H**) IUE (E14) of UbC-EGFP^*flox*^, UbC-Cherry and CreER^T2^ in the absence of the transposase (hyPBase). Without Tx administration, brains processed at P20 displayed green and red-labeled cells. (**I**) IUE (E14) of UbC-EGFP^*flox*^, UbC-Cherry and CreER^T2^ in the absence of transposase (hyPBase). After Tx administration, there were no traces of the GFP protein in brains processed at P20. CreER^T2^ activation by Tx successfully inhibits floxed non-integrated constructs. (**J**) Co-electroporation of CreER^T2^, UbC-EGFP^*flox*^ and UbC-mCherry in the presence of the hyPBase. Genomic integration of EGFP floxed plasmids permanently labels cells with a high rate of division. Green cells, corresponding to glial lineages located within the corpus callosum (cc) and cortical areas. (**K**) Short term labeling following Tx administration one day after IUE (E12) of the UbC-TAU·*StarTrack* mixture and analyzing the brains five days later. (**L**) After UbC-*StarTrack* and UbC-H2B·*StarTrack* IUE (E12), fluorescent reporters remained brightly expressed 8 months later (P240) having administered Tx to pups at P10.

UbC-*StarTrack* is based on the combination of six different fluorescent reporter proteins (XFPs) cloned into integrable, floxed and ubiquitous constructs (Ubiquitin C, UbC): mT-Sapphire, a UV-excitable monomeric GFP mutant XFP with a large stokes shift; mCerulean, the brightest monomeric fluorescent cyan protein; EGFP, a weak dimer enhanced green fluorescent protein; YFP, weak dimer yellow fluorescent protein widely used; Kusabira Orange (mKO), the brightest orange monomer; mCherry, a photostable red monomer. The appropriate spectral separation of these fluorophores allows the independent confocal acquisition of photostable XFPs with minimal overlap (Fig. 1A). These XFPs were designed to be expressed in the cytoplasm, either in the absence of any specific cell signaling (Fig. 1B) or by fusing the microtubule associated TAU protein to the different XFP constructs to facilitate cell identification (UbC-TAU·*StarTrack*: Fig. 1C). In order to obtain more diversity of clonal markers and further increase the possible combinatorial events in a single cell, the XFPs were also fused to histone H2B to promote their nuclear expression (UbC-H2B·*StarTrack*: Fig. 1D). To achieve heritable and stable labeling of the cell progeny, co-electroporation of two additional constructs was necessary, these expressing the hyperactive transposase of the PiggyBac system (hyPBase) and Cre-recombinase fused to a tamoxifen-inducible mutated estrogen receptor (CreER^T2^, Fig. 1E). To attain efficient expression in all neural types, ubiquitous promoters drove the expression of both the hyPBase and Cre enzymes. As such, a mixture of these fluorescent constructs can be co-electroporated and stochastically integrated into each progenitor, generating unique color codes.

#### Stable and heritable targeting strategy

Regarding the division pattern of transfected progenitor, non-integrated constructs might gradually be diluted by successive cell divisions or may be episomally maintained, affecting the clonal code of single-cell progeny. Glial cells divide repeatedly before their final differentiation, leading to the dilution of episomal plasmids reflecting just the fluorescence of integrated constructs. However, electroporated progenitors committed to the neuronal lineage undergo few cell divisions and thus, little dilution of episomal plasmids will occur, interfering with the clonal analysis^17^. To obtain a stable clonal mark, XFPs were cloned within an integrable region flanked by two terminal repeats (TRs) that are recognized by the PiggyBac transposase (Fig. 1F, purple arrows). In addition, to perform an indubitable clonal cell analysis, we inhibited the potential residual episomal plasmids (the copies not integrated by the transposase) using the tamoxifen (Tx) inducible Cre-Lox system. The Cre-recombinase recognizes and cleaves the region flanked by two LoxP-sites strategically inserted into the UbC-*StarTrack* constructs. After Tx administration, Cre cleaves the fluorescent reporter genes flanked by the LoxP-sites in the episomal copies that have not been integrated into the genome (Fig. 1G). When hyPBase driven integration does occur, one LoxP site flanking the XFP is deleted (Fig. 1F) to ensure that CreER^T2^ does not affect the expression of the XFPs incorporated into the host genome.

The efficiency by which the Cre-LoxP strategy prevents the labeling of non-genomic constructs was tested by *in utero* co-electroporation (IUE) of CreER^T2^ in the presence and absence of hyPBase. In addition, a ubiquitous EGFP plasmid (UbC-EGFP^*flox*^) flanked by LoxP sites and a ubiquitous non-floxed and non-integrable mCherry encoding plasmid (UbC-mCherry) were co-electroporated. The UbC-mCherry plasmid was used as a control vector given that it is not integrated into the genome by hyPBase and CreER^T2^ does not inhibit it. After IUE of UbC-EGFP^*flox*^, UbC-mCherry and CreER^T2^ alone, without hyPBase, the plasmids remained as episomal copies. As such, when Tx was not administered, green and red fluorescence was evident in neurons but not in glial cells (Fig. 1H), whereas Tx administration resulted in the lack of green fluorescent labeled cells. Thus, tamoxifen induction of CreER^T2^ prevented the UbC-EGFP^*flox*^expression (Fig. 1I).

Finally, the UbC-EGFP^*flox*^, UbC-mCherry, hyPBase and CreER^T2^ constructs were co-electroporated to confirm that CreER^T2^ activation had no effect on the normal EGFP expression after integration. Ten days after Tx induction, cells with a glial morphology that expressed GFP but not RFP were evident in the corpus callosum of adult brains (Fig. 1J), corroborating hyPBase activity. The presence of green labeled cells (Fig. 1J) in cortical layers indicated that tamoxifen-activated CreER^T2^ had no effect on the expression of integrated UbC-EGFP^*flox*^ constructs, only on the episomal copies (Fig. 1I). Thus, after IUE of UbC-*StarTrack* mixtures and subsequent Tx administration, the fluorophores used were stable and lineage tracing could be performed over either short (4 days post-IUE: Fig. 1K) or longer periods (8 months post-IUE: Fig. 1L).

### UbC-StarTrack *in vitro* and *ex vivo*

**Figure 2:**
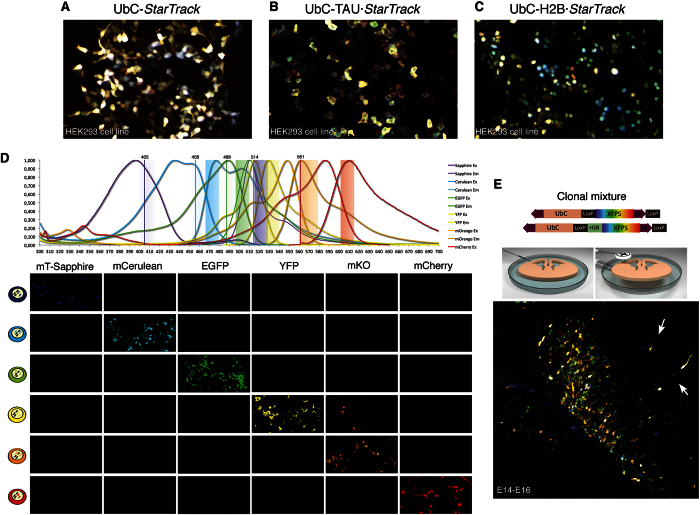
*In vitro* and *ex vivo* transfection of the UbC-StarTrack variants. (**A–C**) Newly generated UbC*-StarTrack* (A), UbC-TAU*·StarTrack* (**B**) and UbC-H2B*·StarTrack* (**C**) constructs were transfected to HEK cells *in vitro* and fluorescent signals were acquired 1 day after transfection. (**D**) Fluorescent spectra of the different fluorescent reporters under the specific confocal acquisition settings. Each fluorescent protein was electroporated individually and its expression was recorded in all confocal channels. Fluorescent expression was only acquired in the designated channel, except for YFP that displayed weak fluorescence in the mKO confocal channel. (**E**) UbC-*StarTrack* and UbC-H2B·*StarTrack* were suitable to perform *ex vivo* clonal analyses after electroporation of the ubiquitous mixture into E14 brain slices since labeled cells migrated out of the area electroporated after 2 days in culture (arrows). *3D-draw provided by Lluis Fortes-Marco.*

To *in vitro* analyze the transfection capability and stability of the different UbC-*StarTrack* variants (Fig. 1B–D) a mixture containing the six XFPs of each group was co-transfected into cultured HEK cells (Fig. 2A–D). Fluorescent reporters signal from each UbC-*StarTrack* mixture were analyzed one-day post-transfection. UbC-*StarTrack* and UbC-TAU·*StarTrack* constructs were uniformly expressed in the cell cytoplasm (Fig. 2A,B). By contrast, UbC-H2B·*StarTrack* reporter gene expression was exclusively expressed in the cell nucleus (Fig. 2C), contributing to the clonal cell identity but without providing morphological information about any cell type. Thus, the six different variant XFP constructs were brightly and stable *in vitro* expressed in the appropriate cell location. Transfections combining different sets of constructs were performed to confirm their co-expression in the same cell at the accurate cell compartment. Moreover, suitable transfection of the different DNA mixtures was obtained in the oligodendroglial precursors cell line Oli-Neu (data not shown).

Each UbC-*StarTrack* construct that expressed a XFP was electroporated into HEK cells independently, to determine the minimal spectrum separation with the confocal acquisition parameters employed. Their spectra led to the ordering of the fluorophores as mT-Sapphire, mCerulean, EGFP, YFP, mKO and mCherry, and they were successfully excited with five different confocal laser lines: 405, 456, 488, 514 and 561 nm. To avoid spectral overlap, emission wavelengths were chosen as close as possible to the peak spectrum, with a range of 10 nm for each XFP. When acquired individually, the electroporated XFPs gave a fluorescent signal in the channel designated to each particular fluorophore (Fig. 2D), and each of the XFPs provided separate fluorescent signals, with the exception of YFP. Indeed, complete spectral separation between YFP and mKO was not possible under the confocal laser conditions employed.

To further determine the viability and stability of these constructs for *ex vivo* experiments, we co-transfected the six UbC-*StarTrack* and the UbC-H2B·*StarTrack* variants into the subventricular zone (SVZ) of embryonic brain slices. After 48h, labeled cells were located within the SVZ, and clonal cells were dispersed throughout the lower cortical layers (Fig. 2E), while some cells appeared to be migrating to other cortical layers (Fig. 2E, arrows).

### *In vivo* analysis of UbC-*StarTrack*

#### Exploring combinatorial possibilities of UbC-StarTrack

**Figure 3:**
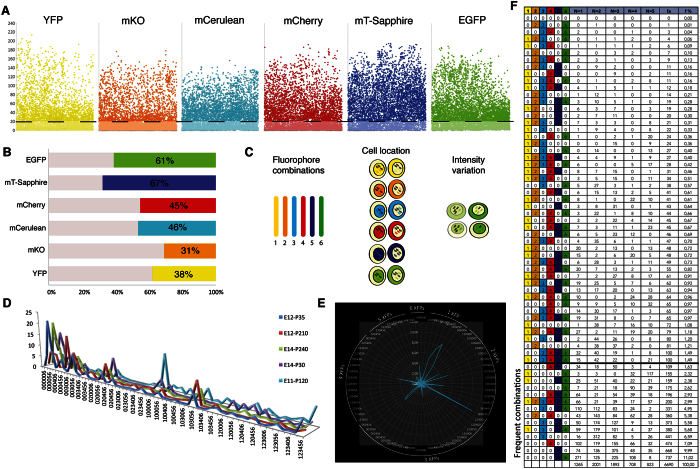
Image analyses of transfected cells. (**A**) Intensity of selected fluorescent reporters within a range of 0–255 in cells analyzed from 8-bit images; a value > 20 was considered to be positive. (**B**) Proportion of positive cells for each fluorescent protein among the 6,690 analyzed (N = 5). (**C**) Combinatorial analysis of the six fluorescent reporters results in 64 possible combinations. Fluorophore location (nuclear vs. cytoplasmic) increases the number of theoretical combinations to 4,096. Since sibling cells must express the fluorophore in the same range of intensity, by simply considering two different groups of intensity the theoretical combinations ascend exponentially to more than 16 million. (**D**) Relative peaks of fluorescent reporter combinations in each individual animal (n = 5). (**E**) Frequency values combining one, two, three, four, five or six fluorophores in the cells analyzed (n = 6,690). (**F**) Raw data analyses showing fluorophore combination per animal. Frequent combinations (at the bottom of the table) were those that less accurately defined sibling cells.

The UbC-*StarTrack* strategy appeared to be a powerful tool to accurately define individual clones, since there are about four thousand theoretical combinations of the 12 fluorescent constructs. To analyze the combinatorial capacity of the XFPs *in vivo*, images from five different IUE animals were analyzed at adult stages (Fig. 3). Since we sought to analyze the combinatorial color possibilities of the construct mixture and the prevalence of XFPs, mice were subjected to IUE on different developmental days from E11 to E14. Adult brains were analyzed with an ImageJ macro designed for this purpose, evaluating 4–6 mosaic images of electroporated areas from each animal. The XFP combinations in 6,690 cells from 5 different animals were analyzed, and clones with a large number of sibling cells (>40) were ruled out in order to prevent clone-size producing a bias in the combinatorial analysis.

Initially, we set out to elucidate the variation in the fluorescence intensity of each fluorophore. The ImageJ macro allowed the minimum threshold values of the fluorescence to be identified, avoiding the inclusion of background noise as a false positive signal in the analysis. Intensity values from 8-bit images were stored in the range 0 to 255 (Fig. 3A) and after sampling the images, the lowest intensity considered to be XFP expression was >20. The XFP with the broadest distribution was mT-Sapphire, exhibiting the widest range of values, while the maximum intensity from the images analyzed corresponded to YFP. The XFP with the lowest fluorescence intensity was mCerulean, with a maximum value of 156, and it was followed by mKO at 178 (Fig. 3A). Both XFPs (mCerulean and mKO) were expressed in an intensity range of 20–50 in more than the 50% of positive cells. In terms of the proportion of cells analyzed that expressed each fluorophore, mT-Sapphire and EGFP were the XFPs most often detected (Fig. 3B), expressed by more than 60% of the 6,690 cells analyzed with a signal. Conversely, mKO was expressed by the lowest number of positive cells, only 30% of those with a fluorescent signal.

We then examined the combinatorial events of the six fluorophores expressed in those 6,690 analyzed cells. Our macro translated these combinations into a six number code, indicating a positive (> = 20) or negative (<20) signal for each fluorophore (Fig. 3C). The order of XFPs in the color code was determined by the confocal acquisition parameters (1-YFP; 2-mKO; 3-mCerulean; 4-mCherry; 5-mTsaphire; 6-EGFP; 0-represented absence of a fluorescent signal). Just considering the six XFPs, UbC-*StarTrack* provided 64 combinations, although the scope of the method was wider as the fluorophores could be located in either the nucleus or cytoplasm, ascending the number of theoretical combinations to 4,096. Moreover, as hyPBase integrates a variable number of plasmid copies, fluorophore intensity was another parameter that should remain within a similar range among sibling cells, exponentially increasing the possible number of combinations. The color-code frequency was performed analyzing the number of times that each 64 possible combination appeared within the macro data. The maximal peak of each combination differed between animals, corroborating the stochastically assignation of color-codes (Fig. 3D). Besides, ordering the codes by linking one, two, three, four, five or the six fluorophores, produced different peaks in specific combinations, the bulk clustered in in combinations of one or two XFPs (Fig. 3E). As hyPBase integrates a variable number of constructs, each combination theoretically has the same probability of being present, yet some combinations were more prevalent than others. Since we analyzed 64 possible fluorophore combinations, a relative frequency around 1.56% indicated equal probability, although 14 combinations of fluorophores had a higher probability than this (Fig. 3F). Single fluorophores or associations of fluorophores with similar spectra (e.g., mCherry-mKO or mT-Sapphire-EGFP) were the most frequent color combinations. The blending of the six XFPs was also very recurrent. Thus, less probable color codes were better candidates to define sibling cells than those with higher probabilities.

#### *In vivo* co-electroporation of UbC-StarTrack

**Figure 4:**
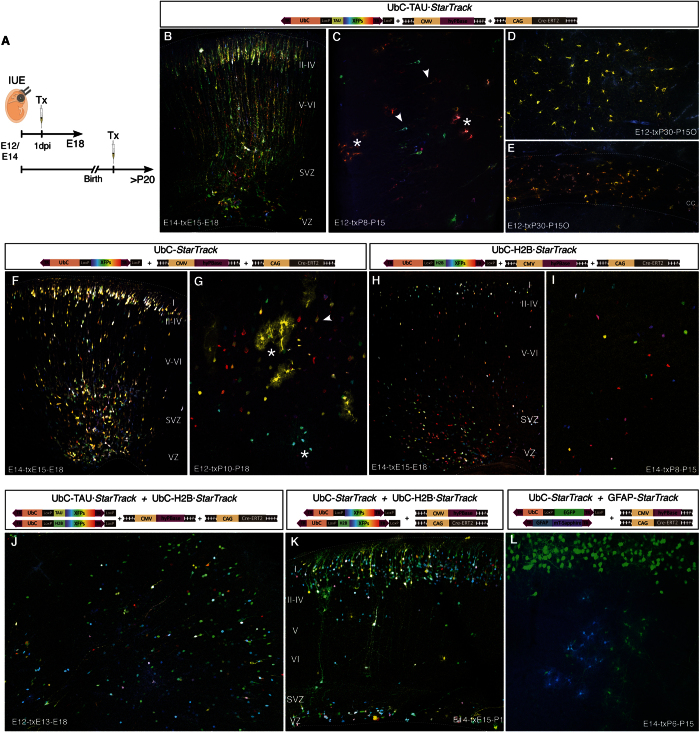
*In vivo* co-electroporation of the UbC-*StarTrack* mixtures. (**A**) Scheme of the procedure. After IUE, tamoxifen (Tx) was administrated either the day after electroporation for perinatal studies or at perinatal stages for brain analyses at adult stages. (**B–C**) IUE of the UbC-TAU*·StarTrack* mixture. (**B**) At E18, immature neurons and radial glial cells were labeled by TAU constructs after Tx administration one day following IUE (E14). (**C**) IUE (E12), Tx (P8), brain analysis (P15): Labeled cells in the piriform cortex with neuronal (arrowheads) and glial (asterisk) morphologies. (**D–E**) IUE (E12), Tx (P30), brain analysis (P150). Large clones of NG2 glial cells occupying several cortical areas (**D**) and oligodendrocytes within the corpus callosum (cc, **E**). (**F–G**) UbC-*StarTrack* mixture. (**F**) IUE (E14), Tx (E15), brain analysis (E18). Cytoplasmic labeling determines the cell fate. (**G**) IUE (E12), Tx (P10), brain analysis (P18). Mature neuronal (arrowhead) and glial (asterisks) cells were identified morphologically. (**H**–**I**) Nuclear UbC-H2B·*StarTrack* mixture labeled only the cell nucleus without providing information regarding cell identity. (**H**) IUE (E14), Tx (E15), brain analysis (E18). Labeled cells indicated the distribution (as in **B**,**F**) but not the cell phenotype. (**I**) IUE (E14), Tx (P8), brain analysis (P15): labeled cells validated the stable expression of those constructs. (**J**) IUE (E12), Tx (E13), brain analysis (E18): combination of the cytoplasmic, UbC-TAU*·StarTrack* and nuclear UbC-H2B*·StarTrack* mixtures. (**K**) IUE (E14), Tx (E15), brain analysis (P1). Co-electroporation of the non-specific cytoplasmic, UbC-*StarTrack* and nuclear UbC-H2B*·StarTrack* mixtures is the best plasmid combination to track sibling cells. (**L**) IUE (E14), Tx (P6), brain analysis (P15). Each ubiquitous-*StarTrack* plasmid could be used individually and/or in combination with other plasmids. Vector expressing mT-Sapphire driven by the GFAP promoter was co-electroporated with the Ubiquitous vector expressing EGFP.

Cell dispersion analyses of the different UbC-*StarTrack* mixtures were performed *in vivo* at both at embryonic and adult stages (Fig. 4A). After IUE of the UbC-TAU·*StarTrack* mixture (Fig. 1C), labeling appeared in both radial glial cells and SVZ progenitors at perinatal stages. Moreover, transfected immature neurons were located in upper and lower cortical layers (Fig. 4B). At adult stages, TAU-fused fluorescent protein was expressed accurately in both neuronal (Fig. 4C, arrowheads) and glial lineages (Fig. 4C, asterisks). Five months after IUE, large glial cell clusters comprised of sibling cells were recognizable by the expression of the same combination of XFPs (Fig. 4D,E). Cells were morphologically identified as either cortical NG2 cells (Fig. 4D) or white matter oligodendrocytes (Fig. 4E). Thus, TAU-fused fluorescent protein proved to be a good option to track and morphologically identify cells. After UbC-*StarTrack* IUE (Fig. 1B), the cytoplasmic expression of XFPs allowed different cell morphologies to be characterized (Fig. 4F,G). At perinatal stages, radial glia cells with their processes contacting the pial surface were evident morphologically and immature neurons occupied upper and lower cortical layers (Fig. 4F), whereas both neurons (Fig. 4G, arrowhead) and glial cells (Fig. 4G, asterisks) were successfully tracked at adult stages. Nuclear constructs (UbC-H2B·*StarTrack*: Fig. 1D) were designed to increase the number of fluorescent labels, resulting in larger combinations of fluorophores to ensure clonal coding. IUE of the plasmid mixture did not allow the morphological identification of cells at either perinatal (Fig. 4H) or adult stages (Fig. 4I). Thus, to elucidate the theoretical increment of XFP combinations necessary for clonal analyses, UbC-H2B·*StarTrack* was combined with either UbC-*StarTrack* or UbC-TAU·*StarTrack*. Co-electroporation of nuclear plasmids with the UbC-TAU·*StarTrack* resulted in a reduction in the TAU-fused fluorescent protein signal (Fig. 4J). Since the nuclear fluorescence was much brighter than the intensity of TAU fluorescence, the confocal conditions limited adequate signal acquisition of both reporters. By contrast, co-electroporation of UbC-*StarTrack* and UbC-H2B·*StarTrack* resulted in successful expression of both reporters, maintaining the cytoplasmic label along with the valuable information provided by the nuclear marker (Fig. 4K). As such, this latter combination was the strategy selected to further define clonally-related cells. Moreover, ubiquitous constructs were co-electroporated with the GFAP-*StarTrack* constructs^15^, in order to probe the extended potential and applications of this tool, combining different expression vectors under the control of different promoters (Fig. 4L).

### UbC-StarTrack to target different cell lineages in distinct brain areas

**Figure 5:**
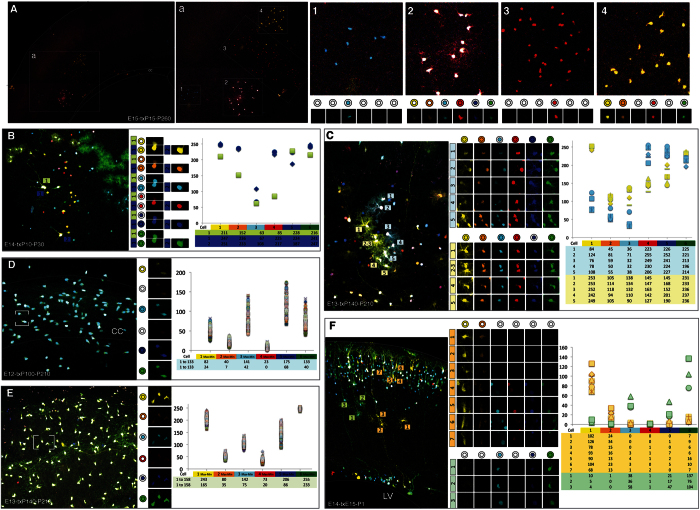
Clonally-related cells from different lineages targeted by IUE of the UbC-*StarTrack* + UbC-H2B·StarTrack. (**A**) Qualitative cell analysis based on the color combination and fluorophore cell location (nuclear or cytoplasmic). (a) UbC-StarTrack labeled cortical clones numbered from 1 to 4. (1–4) Different clonal color-codes detailed by the expression of each fluorescent reporter ordered as: YFP, mKO, mCerulean, mCherry, mT-Sapphire and EGFP. (**B**) Neuronal clone dispersed throughout the cerebral cortex after IUE (E14), Tx (P10) and brain analysis (P30). Fluorescent reporter expression showed one cell (1-green) not clonally-related with the blue clone. Intensity values for each cell are represented on the graph colored and numbered accordingly with their clonal color identity (green or blue). (**C**) Sibling adult astrocytes were located in tighter domains after IUE (E13), Tx (P140), brain analysis (P210). Intensity values for each fluorescent reporter is graphically represented for individual cells of two clones (blue and yellow) comprising five cells each one. (**D**) Oligodendrocytes within the corpus callosum (cc) formed large clusters of clonally related cells after IUE (E12), Tx (P100), brain analysis (P210). Color-code and intensity values for each of the 133 clonally-related cells are dotted in the graph. Both, maximum and minimum intensity values for each fluorescent reporter are detailed in the table. (**E**) NG2 cells occupying several olfactory bulb layers. IUE (E12), Tx (P140), brain analysis (P210). Specific color-code and fluorescent cell intensity for each reporter is represented in the graph. Both, maximum and minimum intensity values of the 158 clonally-related cells are arranged in the table. (**F**) P1 labeled cortical neurons in the dense cortical plate and radial glia cells connecting with the pial surface after IUE (E14) and Tx (E15). Two mixed cell clones formed by immature astrocytes and neurons (orange and green). Note the color-code for the seven clonally-related orange cells and the three green cells. Intensity values for each fluorescent reporter are presented in the graph.

The IUE of nuclear and cytoplasmic UbC-*StarTrack* mixtures allowed glial and neuronal lineages to be tracked. Sibling cells were defined by the same composition of fluorophores, both in the same cell location and range of intensity (Fig. 5). Cells were identified as neurons, astrocytes, oligodendrocytes and NG2 cells according to morphological criteria, and using immunohistochemistry for specific cell type markers (data not shown). To identify clonally-related cells, we performed a qualitative cell analysis based on the color combination and fluorophore cell location (nuclear or cytoplasmic). Different UbC-*StarTrack* labeled cortical clones were easily recognizable by their different fluorescent reporter combinations (Fig. 5Aa). To assess the color-code of each clonal cell, the expression of every fluorophore was individually analyzed (Fig. 5Aa1). Then, to further address the clonal cell identity, we quantified the fluorophore intensity in each cell with the same color-code (Fig. 5B–F). These parameters were necessary to accurately define a clone. Since the first cell generated after IUE were neurons, the inhibition of non-integrated plasmids was critical to obtain the same clonal code for all the neuronal progeny. Neurons expressing the same nuclear and/or cytoplasmic combination of fluorophores in the same range of intensity clearly maintained clonal relationships (Fig. 5B). Indeed, it was possible to observe two nearby cells that were not clonally related (blue and green labels in Fig. 5B), since they differed in terms of the YFP, mKO and mCherry fluorescent proteins locations (nuclear and cytoplasmic vs. only cytoplasmic). Moreover, in this case fluorophore intensity variation between sibling neurons was up to 40-points (Fig. 5B, graph).

While clonally related sister neurons were arranged sparsely and rarely grouped in the same domain (Fig. 5B), glial clones produced a variable number of densely arranged cell clusters depending on the cell lineage (Fig. 5C–E). As such, UbC-*StarTrack* allowed the identification of various clones from different glial lineages, even when were located close together (Fig. 5A,C). The number of clonally related sibling astrocytes (Fig. 5C) was lower than that of oligodendrocytes and NG2 cells (Fig. 5A,D,E). After we defined the fluorophore composition of adult labeled cells, the intensity values established their clonal identity. For example, two different astrocyte clones in Fig. 5C (blue and yellow numbered clones) were discriminated by both fluorophore expression and intensity values (Fig. 5C, graph). Besides, UbC-*StarTrack* allowed to trace the oligodendroglial lineage (Fig. 5D). Large numbers of sibling oligodendrocytes were located within the corpus callosum in adult brains (Fig. 5D). Following IUE with the clonal mixture, NG2 sibling cells formed huge clones throughout adult olfactory bulb layers (Fig. 5E), as described previously with *StarTrack*^16^. In those cases, cell clones were clearly defined not only by color-codes but intensity values (Fig. 5D,E, graphs). Interestingly, the relationship between fluorophores and intensities was likewise maintained within mixed clones formed by cells from different lineages (Fig. 5F). At perinatal stages, immature astrocytes and neurons were clonally-related with the same color-codes and fluorophores intensity values (Fig. 5F yellow and green clones). We highlighted that fluorophore intensity variation was less than 150 points within analyzed sibling cells, independently of their lineage. This demonstrated certain variation in the clonal behavior of neuronal and glial populations and indeed within glial populations, the astrocyte clones were smaller than those of NG2 cells or oligodendrocytes.

**Figure 6:**
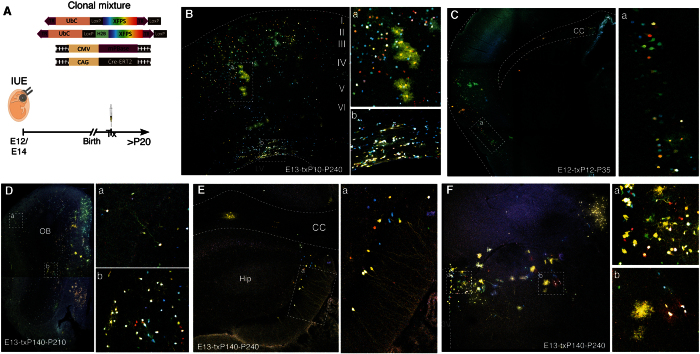
Cell targeting in different brain locations with UbC-*StarTrack* + UbC-H2B·StarTrack. (**A**) Different brain regions targeted after *in utero* co-electroporation of the UbC-*StarTrack* and UbC-H2B·StarTrack mixtures. (**B**) Adult labeled neuronal and glial cells located in the cerebral cortex (a) and within the SVZ (b). IUE (E13), Tx (P10), brain analysis (P240). (**C**) Piriform cortex labeled after IUE at E12, placing the electrodes towards the lateral cortical stream. Glial and neuronal lineages labeled in piriform cortex (a). IUE (E12), Tx (P12), brain analysis (P35). (**D–F**) Targeting extracortical brain areas: (**D**) olfactory bulb (IUE E13, Tx P140, OB analyses P210); (**E**) hippocampus (IUE E13, Tx P140, analyses P240); (**F**) hypothalamus (IUE E13, Tx P140, analyses P240). cc, Corpus callosum; LV, Lateral Ventricle; OB, Olfactory bulb; Hip, hippocampus; 3 V, third ventricle.

UbC-*StarTrack* and UbC-H2B·*StarTrack* co-electroporation (Fig. 6A) tracked neural populations located in diverse brain areas depending on both the orientation of the electrodes and the embryonic stage of targeted progenitors (Fig. 6B–F). Adult neurons were located in their corresponding cortical layers, after E13 electroporation with the positive electrode oriented towards the dorsal pallium (Fig. 6B). Otherwise, glial clones occupied several cortical layers, corpus callosum and SVZ (Fig. 6Ba,b). To trace cell lineages in the adult piriform cortex (Fig. 6C), IUE was performed before E12, placing the electrodes laterally to target the lateral cortical stream. Either neurons or glial cells were labeled in this cortex (Fig. 6Ca). Different extracortical brain areas were labeled after IUE at E13. Positioning the electrodes towards the more rostral area allowed clonal analyses and lineage tracing to be performed in the olfactory bulb (Fig. 6D). However, by injecting the plasmid mixture into the third ventricle and placing the paddles either laterally or ventrally, cells were targeted in the hippocampus (Fig. 6E) and hypothalamus (Fig. 6F) respectively. Thus, the UbC-*StarTrack* strategy allowed several brain regions to be targeted raising enormous possibilities for lineage-tracing studies.

## Discussion

We developed UbC-*StarTrack* to track and compare different clonal populations from the same or different lineages. This tool represents an efficient approach to track the progeny of neural progenitors in diverse brain areas from embryonic to adult stages, irrespective of their mitotic activity. Using UbC-*StarTrack*, precise identification at the single cell level could be achieved through the stochastic genome integration of six different XFPs expressed in specific cell compartments (nucleus and cytoplasm). This produced a unique color code for each parental cell and its descendants, allowing neurons and/or different glial cells from single-fate restricted, bi-and tri-potent precursors to be identified.

The enormous cell heterogeneity of the adult brain will not be totally understood without specific studies to analyze all the progeny of single cells. Transcriptome analyses^18^ or somatic mutations during development^19^,^20^ have proven to be powerful approaches to determine the nature of different neural populations or to establish lineage cell relationships, respectively. Similarly, retroviral vectors have classically been used for tracing cell lineage but since cells must be in cycle for them to integrate stably, the quiescent state of some neural progenitors means they are ineffective to track some populations. More recently, cell lineage approaches have been developed that are based on the probability of labeling by sporadic isolated genomic recombination e.g., in presence of low Tx doses^6^,^21^. However, it is not absolutely clear that the cells labeled by these approaches are from just one progenitor or whether they are from different progenitors that are found nearby in the same region. Moreover, these approaches rely on labeling few cells, impeding further intra-clonal studies to define any physiological and functional relationships.

We previously used the PiggyBac system for long-term clonal tracking of the glial lineage in different brain regions^22^,^23^,^24^. The PiggyBac transposase specifically inserts a sequence into the genome of transfected cells that contains two terminal repeat regions at 5′-TTAA-3′ sites^25^, which in the case of UbC-*StarTrack* included a reporter gene. Here we employed a hyperactive version of the mPBase (hyPBase) that integrates a variable number of transposable copies into the genome at a 10-fold higher transposition rate (9 copies on average), without compromising genomic integrity^26^,^27^. Nevertheless, some electroporated constructs will be transfected into progenitor cells but not integrated into the genome by the hyPBase. Furthermore, the permanent and stable expression of non-integrated constructs in cells that divide little after electroporation has been reported^17^, highlighting the relevance of those non-integrated constructs in clonal methods based on multicolor codes where the combination of different XFPs reflects the progeny of a single cell. Unlike other lineage approaches^12^,^13^, episomal plasmids must be inhibited to establish a unique color code for the entire progeny of a single cell. Indeed, non-integrated or episomal plasmids can generate alterations in the fluorescent clonal code of sibling cells, impeding the accurate analysis of these lineages. To overcome these limitations and to achieve a unique ID for the progeny of an individual cell, we used a Cre-LoxP recombination strategy to silence the expression of non-integrated reporters^28^. Our system was improved by using a tamoxifen-inducible Cre recombinase^29^. Thus, co-electroporation of the UbC*-StarTrack* constructs resolved both the issues related to integration and the inhibition of non-integrated constructs in order to produce a reliable clonal signal in all the progeny, irrespective of the lineage or time of electroporation. To analyze early-generated cell lineages, the tamoxifen administration should be performed 1dpe to accurate label all the cells from single progenitors with the same color-code.

Regarding the evaluation of the clonal markers, the theoretical number of possible combinations after the co-electroporation of both UbC-*StarTrack* and UbC-H2B·*StarTrack* mixtures ascends to more than four thousand. In fact, UbC-*StarTrack* is the clonal method with the widest range of XFPs, expanding the clonal combinations to allow accurate identification of sibling neural cells. While other approaches used Red-Green-Blue reporters^13^,^30^ or four fluorophores^12^, our clonal method was based on the expression of six different reporters. These XFPs were selected on the basis of their photostability and brightness^31^, without requiring immuno-tagging as other clonal approaches^32^. To corroborate the hypothetical UbC-*StarTrack* potential, we estimated the XFP combinations produced in cells from different IUE brains. In terms of individual fluorophore expression, mKO, YFP or Cerulean could be detected in 30–40% of the cells analyzed, indicating they are useful XFPs for the purpose. The less frequent XFPs were relevant as the clones that expressed these reporters are reliable because of their lower probability. Conversely, prevalent XFPs like EGFP or mT-Sapphire are less informative to define cell clones but they are necessary to increase the number of possible combinations and they contribute to defining less probable combinations. According to the data obtained from more than 6,000 cells, some fluorophore combinations accurately define clones and others, as single-fluorophore expression, do not.

Given the high levels of DNA transposition of the PiggyBac transposase^33^, the distinct intensities of the XFP reporters detected for individual cells were important to define clones. While reporter intensity might vary in function of acquisition conditions, neither the confocal laser intensity nor the range emitted by the laser changed within the animals. Only the gain, line/frame average and the offset could be modified when initializing the acquisition of a new animal. Once defined, these parameters were maintained for the acquisition of the whole brain. Moreover, intensity values within cells with the same color-codes did not vary more than 150 points. Together, this information should be taken into consideration when defining sibling cells with UbC-*StarTrack*: fluorophore combination, fluorophore location within the cell and fluorophore intensity.

To achieve the study of all neural cells we used UbC promoter, which drives consistent expression in different cell types^34^ and in our hands, it allowed relationships to be established between sibling cells in all neural populations, making comparisons within or between lineages effortless. Our transposon-based genomic approach enabled us to track the entire progeny, which is not possible with previous methods due to the many cell divisions prior to differentiation^35^,^36^. There were two main differences between glial and neuronal lineages after UbC-*StarTrack* IUE: clonal cell dispersion and the number of cells per clone. Regarding clonal dispersion, sibling neurons were more scattered than glial cells since neurons often migrate to their final position far from their site of birth^37^. After targeting E12 progenitors, neuronal clones are comprised of less than 10 sibling neurons^6^, while clonally related-glial cells were located in adjacent domains since these cells actively divide at their final position^38^. Regarding cell number, glial clones form clusters of 5 to 20 cells in the case of astrocytes, and of more than 400 cells in the case of NG2 cells^39^. UbC-*StarTrack* tracked large oligodendrocyte clones that were recognizable by their morphology and due to their position in the adult corpus callosum. Thus, the distribution of clonally related cells and their cell number was related to their cell lineage.

Together, these data demonstrate the use of this approach to efficiently track the entire progeny of specific single progenitors. Besides being an efficient method for lineage analysis, UbC-*StarTrack* is also a versatile method that is finely tuned and adapted to a broad number of specific variables regarding cell fate tracking. Indeed, we generated 18 ubiquitous, bright and stable XFPs, which can be readily combined with other expression vectors. Moreover, this approach could be adapted to analyze any cell lineage by using appropriate promoters in order to simultaneously track different populations. Moreover, genetically engineered mouse models can be combined with the UbC-*StarTrack* constructs, providing additional information. Indeed, in transgenic mice containing any XFP the loss of a specific fluorescent UbC-*StarTrack* construct will provide significant information. Our floxed constructs are suitable to track clonal related lineages, even in Tx inducible transgenic mice lines. In fact, using such transgenic mice, Tx administration will activate the conditional gene while the non-integrated UbC-*StarTrack* constructs will be inhibited, overcoming the need for the CreER^T2^ plasmid electroporation. Moreover, *in vitro* or *ex vivo* clonal analysis using a wide range of primary cultures could be performed.

Finally, UbC-*StarTrack* will undoubtedly be a useful tool to understand the fundamental events in cancer biology, serving to address the lineage heterogeneity within primary tumors or metastatic niches, to track cancer stem cells and their progeny, and to study the precursor-progeny relationships within tumorigenic cells. Another important area, in which our method could add important information is that of neural disease, providing information as to how cell clonality may be involved in the recovery from injury^40^, or the specific role of clonally related cells in developmental or neurodegenerative brain disorders.

## Methods

### Animals

Wild type C57BL/6 mice from the Cajal Institute animal facility were treated according to the European Union guidelines on the use and welfare of experimental animals (2010/63/EU) and those of the Spanish Ministry of Agriculture (RD 1201/2005 and L 32/2007). Performed experimental approaches were approved by the Cajal Institute, CSIC Animal Experimentation Ethics Committees and the Community of Madrid (Ref.PROEX 44/14). The day of detection of the vaginal plug was defined as the first embryonic day (E0) and the day of birth as postnatal day 0 (P0).

Wild type C57BL/6 mice from the Cajal Institute animal facility were treated according to the European Union guidelines on the use and welfare of experimental animals (2010/63/EU) and those of the Spanish Ministry of Agriculture (RD 1201/2005 and L 32/2007). The CSIC Bioethical Committee approved all the procedures used here. The day of detection of the vaginal plug was defined as the first embryonic day (E0) and the day of birth as postnatal day 0 (P0).

### Construct generation

Oligonucleotides were obtained from SIGMA-ALDRICH, and the PCR products obtained with these were cloned using *CloneJET PCR Cloning Kit* (Fermentas) and cohesive restriction enzymes (Fermentas) in all cases. The Rapid DNA Ligation Kit (FERMENTAS) was used for ligations and the resulting products were transformed into *E. coli* JM107 bacteria using the “*TransformAid”* Bacterial Transformation Kit (Fermentas).

To generate UbC-*StarTrack*, we employed a PiggyBac plasmid encoding the enhanced green fluorescent protein (EGFP) under the control of ubiquitous human Ubiquitin C (UbC) gene promoter, pPB-UbC-EGFP (Yusa *et al*. 2009). Initially, a cloning vector containing the LoxP sites (34 bp) and a multiple cloning site (MCS) was generated. The resulting plasmid, pPB-UbC-MCS^*flox*^ was used successively to clone the XFPs: mT-Sapphire, mCerulean, yellow fluorescent protein (YFP), EGFP, monomeric Kusabira Orange (mKO), and mCherry. The XFPs were amplified by PCR from the pPB-GFAP-XFPs *StarTrack* constructs (García-Marqués-López-Mascaraque, 2013), and the floxed and integrable plasmids expressing six different XFPs were developed, named UbC-*StarTrack*. The sequence encoding histone h2B (H2B) was amplified by PCR using the pPB-GFAP-XFPs *StarTrack* constructs as a template and they were fused to each fluorescent protein in the UbC-*StarTrack* constructs: UbC-H2B·*StarTrack*. Moreover, the Tau protein was amplified from the P3-IRES-tauEGFP plasmid (Adgene #15643) and fused to the different XFPs, generating the UbC-TAU·*StarTrack* constructs. All the constructs were sequenced to assess the efficiency of cloning.

The vector containing the hyperactive transposase of the PiggyBac system (hyPBase) was kindly provided by Prof. Bradley, while the pCAG-CreER^T2^ plasmid was a kind gift from Connie Cepko (Addgene #14797). A non-integrable plasmid encoding mCherry under the UbC promoter (UbC-mCherry) was used as a control of electroporation (Figueres-Oñate *et al*.^17^).

### HEK 293 transfected cells

HEK293 cells were cultured to validate the *in vitro* expression of the constructs generated. Briefly, 1.5–2·10^5^ cells per well were cultured on polyornithine-plated 6-well plates maintained at 37 °C in an atmosphere of 5% CO_2_, and in DMEM (Invitrogen) supplemented with 10% fetal bovine serum (Sigma-Aldrich) and 0.5% penicillin/streptomycin (Invitrogen). Transfection was performed either with the calcium phosphate method or with the commercial transfection reagent TurboFect (Life Technologies). In both cases, a total amount of 3 μgr from the DNA mixture was administrated to each well. The expression of the newly generated constructs was assessed 1 day after transfection. To long-term clonal experiments, cells were passaged every 3–4 days the confluent cultures using 0.05% Trypsin-EDTA (Gibco).

### Electroporation of organotypic slices

After embedding E14 brains in low melting point agarose dissolved in 1X KREBS buffer, 300 μm slices were placed on polycarbonate membranes (Whatman) placed in KREBS medium. UbC-*StarTrack* plasmid mixture was then injected into the SVZ with a borosilicate glass filament. To avoid direct contact of the electrode with the slice, the membranes containing the slices were placed between a protective agarose support, the electrodes were placed in position and then, 2 pulses (voltage: 80 mV, 5 ms on/500 ms off) were delivered to pass the reporters into the cells. After electroporation, membranes containing brain slices were cultured 1 hour in MEM medium (Life Technologies) and then were placed in Neurobasal medium (LifeTechnologies) for long-term cultures. Slices were analyzed after 48–72 h incubation at 37 °C in an atmosphere of 5% CO_2_ in Neurobasal.

### In utero electroporation (IUE)

IUE was performed as described previously (Figueres-Oñate *et al*.^17^). Briefly, E11 to E15 pregnant mice were anesthetized with 1.5% isofluorane/O2 inhalation and their uterine horns were exposed by midline laparotomy. Intraventricular lateral injections into E11-E12 brain embryos were guided by an ultrasound device (VeVo 770; VisualSonics) and embryos from E13 onwards were visualized by trans-illumination. Five consecutive electric square wave pulses (from 28 V, E11 to 37 V, E15; 50 ms duration) were applied to each embryo, after which the uterine horns were replaced into the abdominal cavity. Dams were placed in clean cage to recover and they were monitored closely.

### Tamoxifen administration

Tamoxifen (Tx, Sigma-Aldrich) was dissolved in corn oil (Sigma-Aldrich) at a concentration of 20 mg/ml and a single dose of 5 mg/40 gr body weight was administered. When analyzing embryos, Tx was administrated to pregnant females by intraperitoneal injection 24 h post IUE, whereas to analyze adult mice it was administered at perinatal stages.

### Tissue processing

Mice were analyzed at embryonic, postnatal and adult stages. When embryos were used for analysis, the dams were sacrificed, the embryos were extracted from uterine horns and they were then decapitated. Postnatal mice were anesthetized by hypothermia (P1 to P6) or intraperitoneal injection of sodium pentobarbital (Dolethal, 40–50 mg/Kg: from P6 onwards), their brain was fixed by transcardiac perfusion with 4% paraformaldehyde in 0.1 M phosphate buffer and then post-fixed overnight in the same fixative. Coronal/sagittal serial vibratome sections (50–100 μm thick) were then obtained.

### Image processing and data analyses

Fluorescent labeling was visualized under an epifluorescence microscope (Nikon, Eclipse E600) with the appropriate filter cubes (Semrock): UV-2A (FF01-334/40-25) Cerulean (FF01-405/10), GFP (FF01-473/10), YFP (FF01-520/15), mKO (FF01-540/15), mCherry (FF01-590/20) and Cy5 (FF02-628/40-25). Images were acquired on a Leica TCS-SP5 confocal microscope, capturing the different XFPs in separate channels. The wavelength of excitation (Ex) and emission (Em) for each XFP were (in nanometers, nm): mT-Sapphire (Ex: 405; Em: 520–535), mCerulean (Ex: 458; Em: 468–480), EGFP (Ex: 488; Em: 498–510), YFP (Ex: 514; Em: 525–535), mKO (Ex: 514; Em: 560–580), mCherry (Ex: 561; Em: 601–620), and Alexa 633 (Ex: 633; Em: 650–760). Confocal laser lines were in-between 25–40% in all cases and the maximum projection images were created using confocal (LASAF Leica) and NIH-ImageJ software.

The imaging data was analyzed with a custom macro integrated into ImageJ (NIH). The critical step in the macro analysis was to appropriately select the labeled cells for which the threshold for every image in each confocal channel was adjusted in order to create positive cell selection for each XFP. Background subtraction was employed before the threshold selection by applying a smooth filter to improve cell signal selection and a binary image was then created to generate the selection. A watershed filter was applied after the binary image to separate contiguous tagged cells and to analyze them as individual points. Once the selection had been performed, minimal fluorescence intensity was considered as a positive value. Subsequently, the data were automatically organized in a table indicating the maximal and minimal XFP intensity value for each labeled cell and its specific color code represented in the potential clonally related cells. Further analysis was performed to determine sibling cells based on the fluorescence intensity ranges and the location of the fluorophore (nucleus or cytoplasm).

## Additional Information

**How to cite this article**: Figueres-Oñate, M. *et al*. UbC-*StarTrack,* a clonal method to target the entire progeny of individual progenitors. *Sci. Rep.* **6**, 33896; doi: 10.1038/srep33896 (2016).
